# Plantaginis Semen Ameliorates Hyperuricemia Induced by Potassium Oxonate

**DOI:** 10.3390/ijms25158548

**Published:** 2024-08-05

**Authors:** Tian Liu, Liting Wang, Li Ji, Leixin Mu, Kaihe Wang, Guang Xu, Shifeng Wang, Qun Ma

**Affiliations:** Key Laboratory of TCM-Information Engineer of State Administration of TCM, School of Chinese Materia Medica, Beijing University of Chinese Medicine, Beijing 100029, China; liutian_bucm@163.com (T.L.); 18810616290@163.com (L.W.); 18340077215@163.com (L.J.); muleixin123@163.com (L.M.); wangkaihe0201@163.com (K.W.); xu18810536768@126.com (G.X.)

**Keywords:** Plantaginis semen, hyperuricemia, UPLC-QE-Orbitrap-MS, RNA-seq, PPAR signaling pathway

## Abstract

Plantaginis semen is the dried ripe seed of *Plantago asiatica* L. or *Plantago depressa* Willd., which has a long history in alleviating hyperuricemia (HUA) and chronic kidney diseases. While the major chemical ingredients and mechanism remained to be illustrated. Therefore, this work aimed to elucidate the chemicals and working mechanisms of PS for HUA. UPLC-QE-Orbitrap-MS was applied to identify the main components of PS in vitro and in vivo. RNA sequencing (RNA-seq) was conducted to explore the gene expression profile, and the genes involved were further confirmed by real-time quantitative PCR (RT-qPCR). A total of 39 components were identified from PS, and 13 of them were detected in the rat serum after treating the rat with PS. The kidney tissue injury and serum uric acid (UA), xanthine oxidase (XOD), and cytokine levels were reversed by PS. Meanwhile, renal urate anion transporter 1 (*Urat1*) and glucose transporter 9 (*Glut9*) levels were reversed with PS treatment. RNA-seq analysis showed that the PPAR signaling pathway; glycine, serine, and threonine metabolism signaling pathway; and fatty acid metabolism signaling pathway were significantly modified by PS treatment. Further, the gene expression of *Slc7a8*, *Pck1*, *Mgll*, and *Bhmt* were significantly elevated, and *Fkbp5* was downregulated, consistent with RNA-seq results. The PPAR signaling pathway involved *Pparα*, *Pparγ*, *Lpl*, *Plin5*, *Atgl*, and *Hsl* were elevated by PS treatment. URAT1 and PPARα proteins levels were confirmed by Western blotting. In conclusion, this study elucidates the chemical profile and working mechanisms of PS for prevention and therapy of HUA and provides a promising traditional Chinese medicine agency for HUA prophylaxis.

## 1. Introduction

Hyperuricemia (HUA) is a metabolic syndrome resulted from purine metabolism disorders or uric acid (UA) metabolism abnormalities, and it is considered as a risk factor of renal diseases [1,2]. Conventional therapies encompass lifestyle interventions and pharmacological treatments; allopurinol, febuxostat, benzbromarone, and losartan are commonly used [3]. However, these uric-lowering drugs often exhibit side effects including liver and kidney function impairment and skin adverse effects and could lead to a relapse upon withdrawal of the drug [4,5,6]. Given the complexity of hyperuricemia pathogenesis, it is imperative to search for safe, effective, and working mechanism well-defined natural drug for therapy of HUA. 

Plantaginis semen, the dried ripe seed of *Plantago asiatica* L. or *Plantago depressa* Willd., is a damp-draining diuretic and has been extensively used for HUA. According to the Chinese pharmacopoeia, its major function is described as disinhibiting water, penetrating dampness, and removing phlegm, and it can be used for pyretic stranguria, edema, damp-heat diarrhea, swelling and pain of eyes, and phlegm-heat cough [7]. According to the principles of traditional Chinese medicine, the key to preventing HUA lies in invigorating the spleen and eliminating dampness, lowering turbidity, and removing phlegm. In recent years, increasing studies reported the preventive and therapeutic effects of PS on HUA. PS increased urine volume excretion and urine electrolyte excretion [8]; deregulated the expression of TGF-β1, TNF-α, and IL-1β; and reduced the content of urinary protein and UA [9]. Psyllium polysaccharide ameliorated renal damage in rats with gouty nephropathy by regulating the NOD-like receptor protein 3 (NLRP3) inflammasome signal pathway [10]. HUA is usually associated with dysregulation of lipid metabolism [11]. In our previous study, 12 biomarkers were identified from the serum samples of HUA patients, L-tyrosine, L-phenylalanine, arachidonic acid, linoleic acid, oleic acid, stearic acid, LysoPC (18:0), LysoPC (18:1 (9Z)), and LysoPC (16:0) were downregulated in the serum of HUA patients [12]. The metabolomics analyses showed that PS was intimately involved in lipid metabolism, in which 13 differentially expressed metabolites (DEMs) were included, and the glycerophospholipid metabolism pathway was affected [13]. However, the active ingredients of PS were insufficiently elucidated, but the study on the pharmacodynamic mechanism related to HUA was clarified. Therefore, we tried to combine more techniques and methods to explore the active constituents and the mechanisms of PS for prevention and therapy of HUA.

In recent years, the RNA sequencing (RNA-seq) technique has been widely employed to investigate diverse mechanisms of various medicines, and the application of ultra-high-performance liquid chromatography–tandem quadrupole electrostatic field–orbital trap–mass spectrometry (UPLC-QE-Orbitrap-MS) has significantly enhanced its capacity for analyzing chemical constituents in complex systems, especially in traditional Chinese medicine [14]. Therefore, systematic analysis and identification of the chemical components of PS both in vitro and in vivo, along with the exploration of differentially co-expressed genes and various pathways, are crucial for uncovering pharmacodynamic mechanisms and have great importance for clinical applications.

This study employed UPLC-QE-Orbitrap-MS and RNA-seq to identify the components of PS in vitro and in vivo, searching for differentially co-expressed genes, key pathways, and crucial targets. Moreover, RT-qPCR and Western blotting experiments were performed for gene expression confirmation, providing a foundational basis for further research on its mechanisms and clinical application.

## 2. Results

### 2.1. Clarification of Constituents in PS by UPLC-QE-Orbitrap-MS

**Figure 1:**
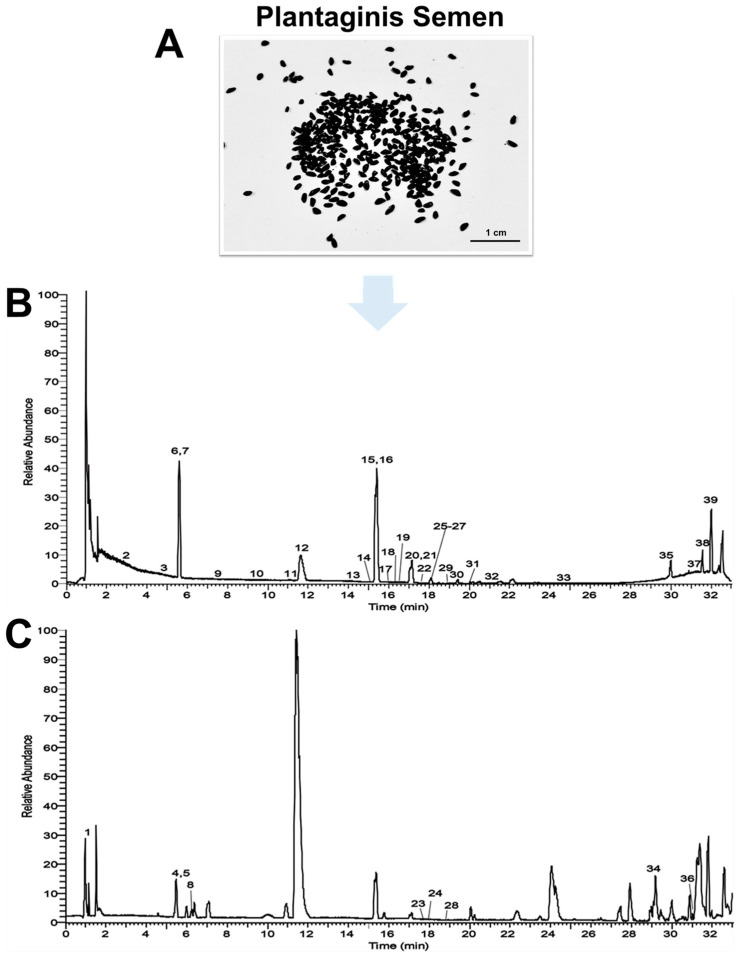
Qualitative analysis of PS extract by UPLC-QE-Orbitrap-MS. (**A**) Photograph of PS (by Tian Liu). (**B**) Base peak ion current of PS extract analyzed in negative-ion mode. (**C**) Base peak ion current of PS extract analyzed in positive-ion mode. (The numbers on the peaks represent the corresponding components in Table 1). The PS extract was filtered with 0.22 μm and then subjected to UPLC-QE-Orbitrap-MS analysis.

**Figure 2:**
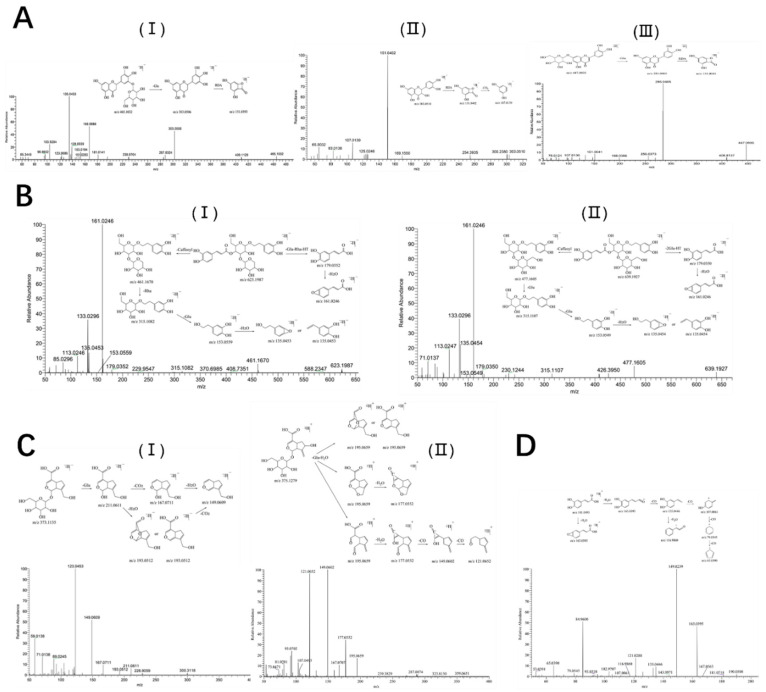
Mass spectrometry fragmentation information and speculated cleavage pathways. (**A**) Identification of flavonoids: I. plantagoside; II. taxifolin; III. cynaroside. (**B**) Identification of phenyl ethanol glycosides: I. acteoside; II. plantainoside D. (**C**) Identification of iridoids: I. geniposidic acid; II. gardoside. (**D**) Identification of organic acids: caffeic acid.

A total of 39 compounds were identified from PS extract, 30 of which were in negative-ion mode (Figure 1A), and 9 were supplemented in positive-ion mode (Figure 1B); the details are listed in Table 1. 14 flavonoids were identified from the PS extract. Taking peak 12, peak 14, and peak 18 as examples, they were identified by the negative-ion mode; the [M-H]^−^ ion of peak 12 at *m*/*z* 465.1042 lost glucose to generate the aglycone parent ion [M-H-Glu]^−^ at *m*/*z* 303.0506. The fragment ion at *m*/*z* 151.0393 was generated by reverse Dills–Alder (RDA) cleavage of the flavonoid parent nucleus. Plantagoside was inferred from the fragments reported in the literature [15,16] and its mass spectrometry fragment information and possible cleavage pathways in negative-ion mode are shown in Figure 2A. The [M-H]^−^ ion of peak 14 was at *m*/*z* 303.0514, its fragments ion at *m*/*z* 151.0402 [M-H-C_8_H_8_O_3_]^−^ was due to RDA cleavage of the flavonoid nucleus, and the fragments ion at *m*/*z* 107.0139 [M-H-C_8_H_8_O_3_-CO_2_]^−^ was formed after losing 42 Da (CO_2_). Compared with the literature [15], it was identified as taxifolin (Figure 2A). The [M-H]^−^ ion of peak 18 was at *m*/*z* 447.0941 due to lost glucose, generating the aglycone parent ion [M-H-Glu]^−^ at *m*/*z* 285.0405. In addition, RDA cleavage occurred in the C ring, resulting in fragment ions at *m*/*z* 151.0041. This was identified as cynaroside (Figure 2A) based on the mass spectrometry fragment information and related literature [17].

Nine phenyl ethanol glycosides were identified in PS, eight of which were identified in negative-ion mode and one in positive-ion mode. The identification process of phenyl ethanol glycosides is shown in Figure 2B. The [M-H]^−^ ion of peak 15 at *m*/*z* 623.2000 lost caffeic acid to generate the fragment ion [M-H-C_9_H_6_O_3_]^−^ at *m*/*z* 461.1670 and continued to lose rhamnose (Rha) to obtain the fragment ion [M-H-C_9_H_6_O_3_-Rha]^−^ at *m*/*z* 315.1082. The fragment ions at *m*/*z* 179.0352, 161.0246, 153.0559, and 135.0453 were the caffeic acid fragment ions, ions of caffeic acid after losing H_2_O, phenyl ethanol aglycone fragment ions, and ions of it after losing H_2_O. It was identified as acteoside, according to the retention time and the fragments of standard substance (Figure 2B).

The [M-H]^−^ ion of peak 19 at *m*/*z* 639.1954 lost caffeic acid to generate the fragment ion [M-H-C_9_H_6_O_3_]^−^ at *m*/*z* 477.1605, continued to lose glucose to obtain the fragment ion [M-H-C_9_H_6_O_3_-Glu]^−^ at *m*/*z* 315.1107, and lost another glucose to obtain the phenyl ethanol aglycone fragment ion [M-H-C_9_H_6_O_3_-2Glu]^−^ at *m*/*z* 153.0549; a fragment ion [M-H-C_9_H_6_O_3_-2Glu-H_2_O]^−^ at *m*/*z* 135.0454 was generated after losing H_2_O. The fragment ions at *m*/*z* 179.0350 and 161.0246 were the caffeic acid fragment ions and ions of caffeic acid after losing H_2_O, respectively. Compared with the fragment information and the related literature [16], it was identified as plantainoside D (Figure 2B).

Four iridoids were identified in PS, two of which were identified in negative-ion mode and two in positive-ion mode. The identification process is demonstrated in Figure 2C. The [M + H]^+^ ion of peak 4 at *m*/*z* 375.1279 lost glucose and H_2_O to generate the fragment ion [M + H-Glu-H_2_O]^+^ at *m*/*z* 195.0659 and continued to lose H_2_O to obtain the fragment ions [M + H-Glu-2H_2_O]^+^ at *m*/*z* 177.0552, which lost CO twice to generate *m*/*z* 149.0602 [M + H-Glu-2H_2_O-CO]^+^ and *m*/*z* 121.0652 [M + H-Glu-2H_2_O-2CO]^+^. Compared with the database and the related literature [16], it was identified as gardoside (Figure 2C). The [M-H]^−^ ion of peak 6 at *m*/*z* 373.1135 lost glucose to generate the fragment ion [M-H-Glu]^−^ at *m*/*z* 211.0611, and it continued to lose H_2_O to obtain the fragment ion [M-H-Glu-H_2_O]^−^ at *m*/*z* 193.0512, lose CO_2_ to obtain the fragment ion [M-H-Glu-CO_2_]^−^ at *m*/*z* 167.0711, or lose H_2_O and CO_2_ at the same time to obtain fragment ion [M-H-Glu-CO2-H_2_O]^−^ at *m*/*z* 149.0609. It was identified as geniposidic acid by comparing the retention time and the fragments of the standard substance (Figure 2C).

A total of three organic acid compounds were identified, two of which were identified in negative-ion mode and one in positive-ion mode (Figure 2D). The [M + H]^+^ ion of peak 34 at *m*/*z* 181.0493 lost H_2_O to generate the fragment ion [M + H-H_2_O]^+^ at *m*/*z* 163.0395, continued to lose CO to obtain the fragment ions [M + H-H_2_O-CO]^+^ at *m*/*z* 135.0446, then lost H_2_O to generate the fragment ion [M + H-2H_2_O-CO]^+^ at *m*/*z* 116.9860. The ions at *m*/*z* 107.0861, 79.0545, and 65.0390 were the C_7_H_7_O, C_6_H_7_, and C_5_H_5_ fragments generated by the fracture. This peak was identified as caffeic acid (Figure 2D), compared with the database and the related literature [17].

### 2.2. Identification of Blood-Migrating Constituents in PS by UPLC-QE-Orbitrap-MS

The bioactive molecules or metabolites produced during drug metabolism pass through the blood and are distributed throughout the body to play pharmacological roles [18]. The blood-migrating constituents in PS are potential anti-HUA effective components of PS. These components influence the synthesis and elimination of uric acid in the body through various mechanisms, thereby ultimately affecting blood uric acid levels.

By comparing the plasma of the control group (group A) and PS administration group (group B) and analyzing the retention time, mass spectrum fragment information, and cracking rule of PS extract and standard substances, 40 components were screened and identified in blood, and 13 of them were prototype components (Table 2). These components maintain a certain concentration in the blood, which may be the potential medicinal ingredient in anti-HUA formulations. 

### 2.3. Plantaginis Semen Ethanol Extract Reversed Rat Urine Acid, Cytokines Levels, and mRNA Expression of Uric Acid Transporter Induced by Potassium Oxonate 

**Figure 3:**
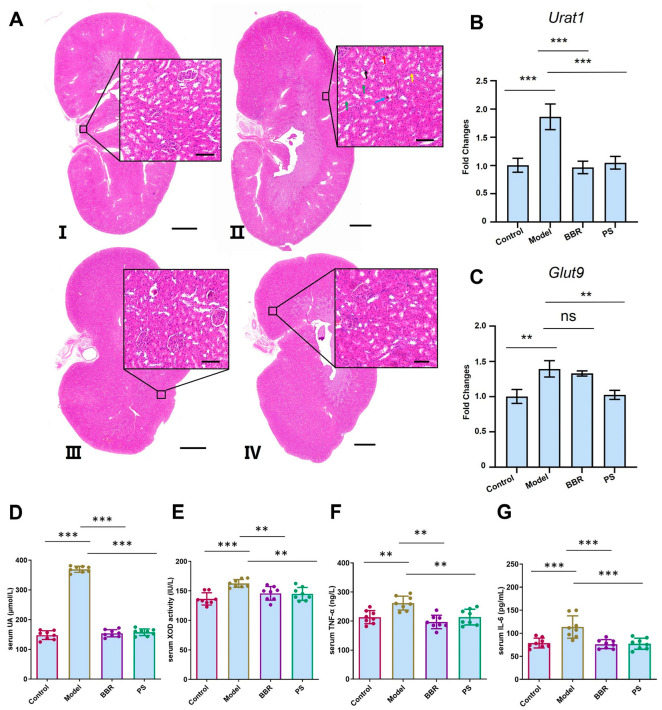
PS significantly reversed histopathologic status, blood biochemical indexes, and mRNA expression of uric acid transporter of HUA rats. (**A**) Representative H&E staining histopathological images of potassium oxonate-induced rat kidney tissue injury treated with or without PS; the blue arrow indicates urate crystals, the yellow arrow indicates inflammatory infiltration, the red arrow indicates lobulated glomeruli, the green arrow indicates renal tubules changed into protein tubular type, and the black arrow indicates renal tubule atrophy. I. Control group. II. Model group. III. BBR group. Ⅳ. PS group. Scale bars for original images: 2000 μm, scale bars for enlarged images: 100 μm. (**B**) mRNA expression levels of *Urat1* and (**C**) mRNA expression levels of *Glut9*. (**D**) UA level, (**E**) XOD activity, (**F**) TNF-α level, and (**G**) IL-6 level. Values represented mean ± SD; one-way ANOVA was used for statistical analysis (** *p* < 0.01 and *** *p* < 0.001). ns: not significant.

In the normal group, the glomeruli and renal tubules showed regular morphology and structure, with no urate crystallization, mesangial hyperplasia, and glomerular segmentation, and no inflammatory cell infiltration was observed. In the potassium oxonate-stimulated HUA model group, urate crystals accumulated in the renal tubules, and protein tubules, mesangial hyperplasia, and glomerular segmentation were observed, accompanied by increased inflammatory cell infiltration. Compared to the model group, the BBR and PS treatment improved renal tubule injury, urate crystal deposition, and inflammatory cell infiltration (Figure 3A).

HUA is characterized by elevated levels of uric acid in the blood. Uric acid in the blood has two main sources: the majority is produced endogenously through purine metabolism, and the rest comes from intake of purine-rich foods in the diet [19]. The results of biochemical indexes showed that the serum UA levels (Figure 3D) and the activity of serum XOD (Figure 3E) of the model group were significantly higher than those of the control group, and the kidneys of the rats in the model group showed obvious pathological changes, indicating that the HUA rat model was successfully developed. 

The level of serum UA of BBR group and PS group significantly decreased, indicating PS could lower serum UA level in potassium oxonate induced HUA rats (Figure 3D). Similarly, PS could lower XOD activity (Figure 3E) and TNF-α (Figure 3F) and IL-6 (Figure 3G) levels in potassium oxonate-induced HUA rats. XOD is an aerobic dehydrogenase that can catalyze the oxidation of xanthine to uric acid [20]. The decrease in XOD activity indicated that PS inhibited the conversion of purine to uric acid and affected the production of uric acid, playing a role in lowering uric acid. Inflammatory responses triggered by HUA-related factors, such as excessive urate crystal deposition and mitochondrial reactive oxygen species, are thought to play a critical role in the pathogenesis of HUA. The pro-inflammatory mediators TNF-α and IL-6 were found to be at high levels and involved in HUA progression [21]. 

Uric acid excretion disorders account for 90% of HUA cases [22]. Uric acid excretion is mainly related to the function of uric acid transporters in the kidneys [23]. *Urat1* and *Glut9* are uric acid reabsorption transporters that play important roles in uric acid excretion; many uric acid-lowering drugs are *Urat1* inhibitors, such as benzbromarone, probenecid, and lesinurad [22,24]. The results showed that the mRNA expression levels of *Urat1* (Figure 3B) and *Glut9* (Figure 3C) in the renal tissues of the model group were significantly higher (*p* < 0.01 or *p* < 0.001) than the control group. PS downregulated the mRNA expressions of *Urat1* and *Glut9* in potassium oxonate-induced HUA rats, which further proves the pharmacological effect of PS in lowering uric acid and the mechanism of PS on the excretion of uric acid by regulating the uric acid transporter in the kidney.

### 2.4. Differential Expression Genes and Related Pathways Analyzed by RNA Sequencing

**Figure 4:**
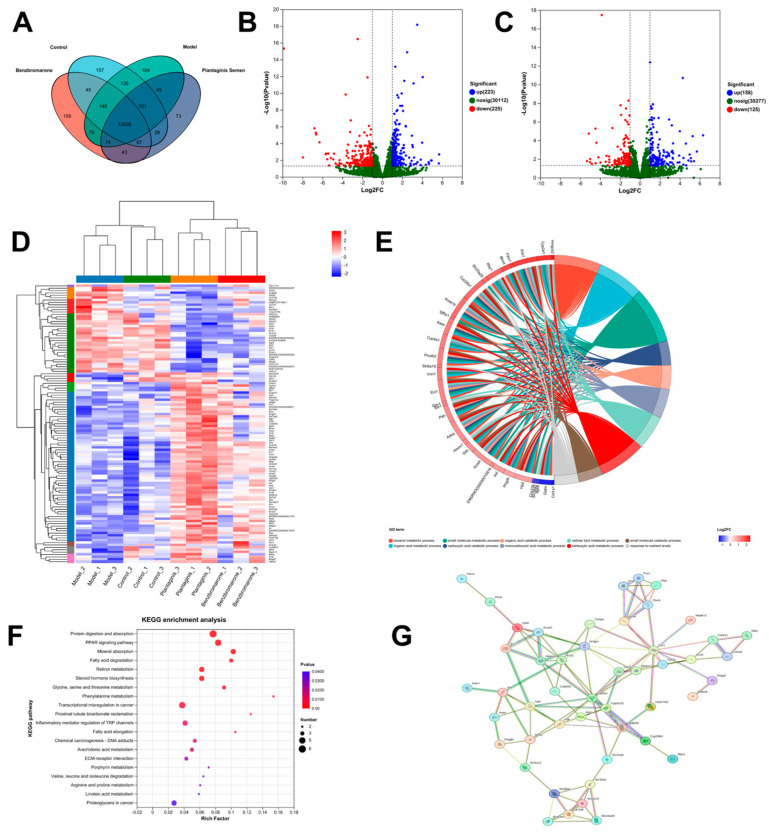
Gene expression profile in PS-treated potassium oxonate-induced HUA rats. (**A**) Venn mapping of altered genes in control, model, BBR, and PS groups in rat kidney tissues. (**B**) Volcano plots of upregulated and downregulated DEGs between PS and model groups. (**C**) Volcano plots of upregulated and downregulated DEGs between model and control groups. (**D**) Heatmap for hierarchical cluster analysis of DEGs between the samples. (**E**) GO and (**F**) KEGG analysis of DEGs in the PS vs. model groups. (**G**) Protein–protein interaction (PPI) networks diagram of DEGs in the PS vs. model groups.

In order to explore the working mechanism of PS on HUA and prove the efficacy of PS against HUA, Transcriptomes from the control, model, BBR, and PS group rat kidney tissues were sequenced. Clean reads on average were obtained from twelve samples. In all clear reads, the Q30 ratio of clean reads was higher than 94.27%, indicating high reliability of the sequencing results. The transcripts and unigenes totaled 38,657 and 20,403, respectively. The Venn analysis diagram between samples is shown in Figure 4A. A total of 2147 differentially expression genes (DEGs) were identified. Overall, 223 upregulated and 225 downregulated DEGs were identified in the PS compared to the control groups, and 158 upregulated and 125 downregulated DEGs were identified in the model compared to the control groups (Figure 4B,C). Hierarchical clustering analysis of the DEGs among four groups (Figure 4D) indicates that PS significantly upregulate the expression of *Slc7a8, Pck1, Mgll,* and *Bhmt* and downregulate the expression of *Fkbp5*, and it has effects on genes related to amino acid metabolism and lipid metabolism. 

GO function enrichment analysis (Figure 4E) showed that compared to the model group, the DEGs in the PS group were mainly enriched for the oxoacid metabolic process, organic acid metabolic process, amino acid transmembrane transporter activity, lipid metabolic process, etc. KEGG analysis showed that the differential genes between the PS and the model group were enriched for 166 pathways, including protein digestion and absorption, PPAR signaling pathway, fatty acid degradation, retinol metabolism, steroid hormone biosynthesis, glycine and serine and threonine metabolism, phenylalanine metabolism, proximal tubule bicarbonate reclamation, inflammatory mediator regulation of TRP (transient receptor potential) channels, fatty acid elongation, etc. The top 20 pathways were enriched and suggested that PS for HUA is predominantly involved in the regulation of amino acids and lipid metabolism as well as in proximal tubule bicarbonate reclamation and inflammatory responses (Figure 4F).

### 2.5. Differential Expression Genes Were Verified by RT-qPCR

**Figure 5:**
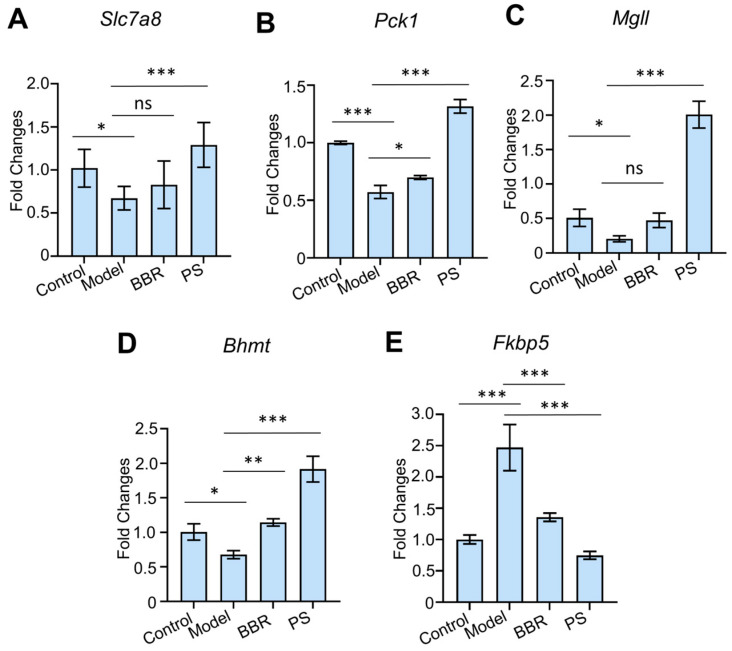
Effects of PS on mRNA expression. (**A**) mRNA expression levels of *Slc7a8*. (**B**) mRNA expression levels of *Pck1*. (**C**) mRNA expression levels of *Mgll*. (**D**) mRNA expression levels of *Bhmt*. (**E**) mRNA expression levels of *Fkbp5*. Error bars indicate SD. One-way ANOVA was used for comparing statistical significance between different groups (* *p* < 0.05, ** *p* < 0.01 and *** *p* < 0.001). ns: not significant.

RNA-seq data suggested multiple key differential expression genes regulated by PS in the treatment of HUA. The DEGs were verified using RT-qPCR, and the trends were consistent with the RNA-seq (Figure 5). The RT-qPCR results showed that the mRNA expression levels of *Slc7a8*, *Pck1*, *Mgll*, and *Bhmt* in the renal tissues of the model group were significantly lower (*p* < 0.001 or *p* < 0.05) than the control group, and *Fkbp5* in the model group were significantly higher (*p* < 0.001) than the control group. *Slc7a8*, *Pck1*, *Mgll*, and *Bhmt* are associated with the regulation of metabolic homeostasis in the treatment of metabolic syndrome [25,26,27,28], and *Fkbp5* plays a crucial role in the inflammatory response [29]. PS upregulated the mRNA expressions of *Slc7a8*, *Pck1*, *Mgll*, and *Bhmt*, while it downregulated the mRNA expressions of *Fkbp5* in potassium oxonate-induced HUA rats, indicating that PS ameliorates HUA induced by potassium oxonate. However, the specific mechanism needs to be further studied. 

### 2.6. PS Regulates PPAR Signaling Pathway

**Figure 6:**
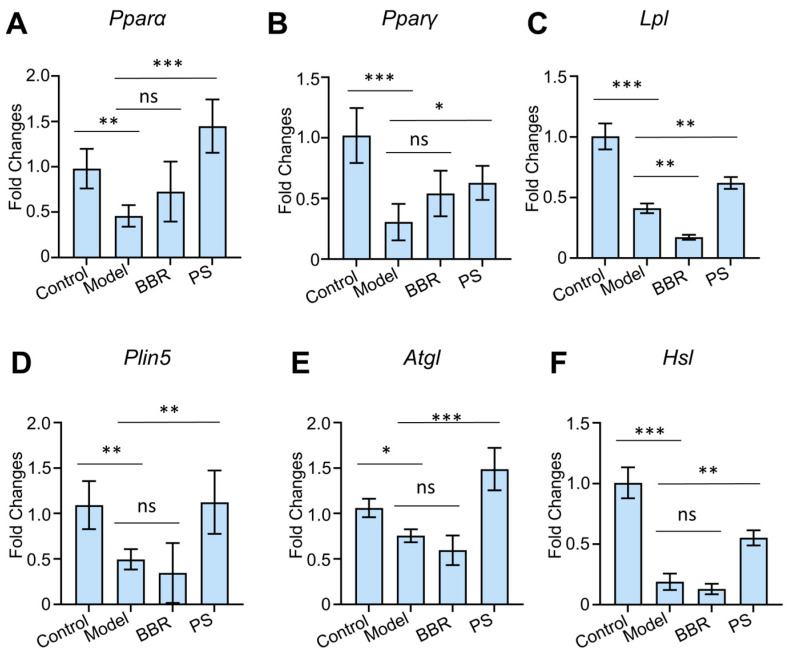
Effects of PS on rat renal tissue mRNA expression associated with PPAR signaling pathway. (**A**) mRNA expression levels of *Pparα*. (**B**) mRNA expression levels of *Pparγ*. (**C**) mRNA expression levels of *Lpl*. (**D**) mRNA expression levels of *Plin5*. (**E**) mRNA expression levels of *Atgl*. (**F**) mRNA expression levels of *Hsl*. Error bars indicate SD. One-way ANOVA was used for comparing statistical significance between different groups (* *p* < 0.05, ** *p* < 0.01 and *** *p* < 0.001). ns: not significant.

**Figure 7:**
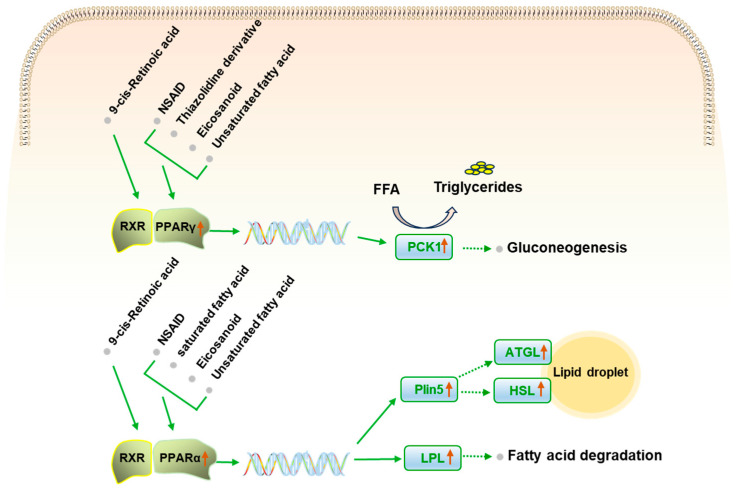
Illustration of the most significant regulated target genes related to PPAR signaling pathway. The vertical red arrows indicate the upregulated trend by PS.

In order to further explore the pathway regulated by PS regarding HUA, the effects of PS on mRNA expression associated with PPAR signaling pathway were explored using RT-qPCR (Figure 6). The result shows that PS upregulated the mRNA expressions of *Pparα*, *Pparγ*, *Lpl*, and *Plin5* in the PPAR signaling pathway (*p* < 0.05) and upregulated expression of *Hsl* and *Atgl*, the downstream genes of *Plin5* (*p* < 0.01). The results further prove the effect of PS on lipid metabolism (Figure 7). 

### 2.7. PS Regulates URAT1 and PPARα Proteins Expression

**Figure 8:**
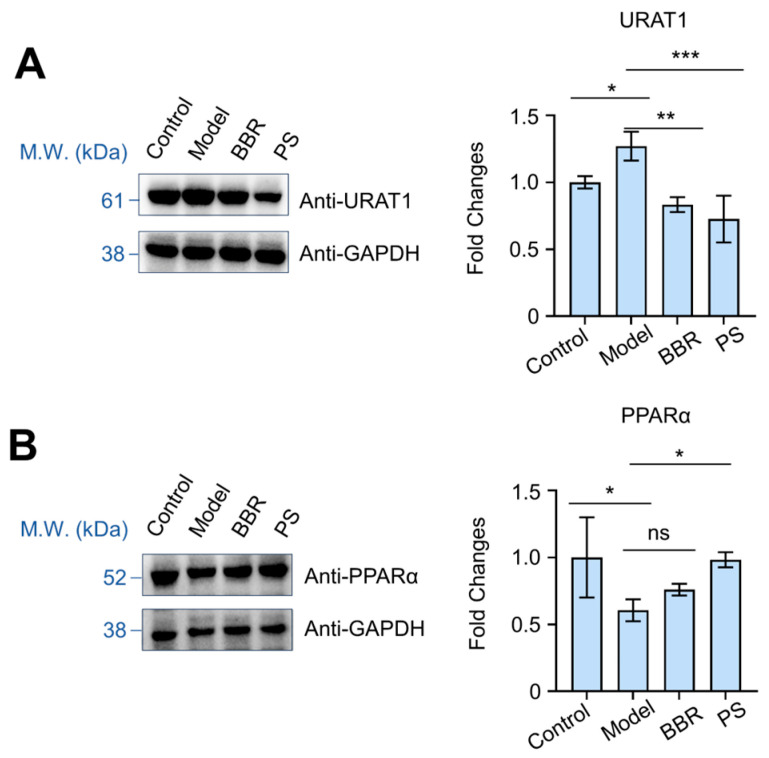
Effects of PS on URAT1 and PPARα proteins expression. (**A**) Representative immunoblots of URAT1 and URAT1 protein levels analysis. (**B**) Representative immunoblots of PPARα and PPARα protein levels analysis. n = 3. Error bars indicate SD. One-way ANOVA was used for comparing statistical significance between different groups (* *p* < 0.05, ** *p* < 0.01 and *** *p* < 0.001). ns: not significant.

To further confirm the working mechanism of PS on regulating uric acid homeostasis, we investigated the effects of PS on the expression of URAT1 and PPARα. Western blot analysis showed that PS administration significantly downregulated kidney URAT1 and upregulated PPARα levels (Figure 8A,B). Compared to the normal control group, the URAT1 expression level was significantly increased (*p* < 0.05), and BBR or PS treatment reversed the elevated URAT1 (Figure 8A). Compared to the normal control group, PPARα expression level was significantly decreased (*p* < 0.05), and BBR or PS treatment upregulated PPARα levels (Figure 8B). URAT1 is a transporter responsible for uric acid reabsorption in renal tubules and is a key target for therapy of HUA [30]. Many uric acid-lowering drugs are URAT1 inhibitors. Inhibition of URAT1 reduces uric acid reabsorption and promotes uric acid excretion [23]. The transcriptional analysis suggests that the PPAR pathway is intimately related to PS’s activity against HUA, and here, the activation of PPARα further confirmed the key roles of the PPAR pathway involved in the activity of PS [31]. These observations clarify the working mechanism of PS, as shown by Western blot.

## 3. Discussion

As a traditional Chinese medicine, PS has been widely used in the clinical treatment of HUA, while the active ingredients and the working mechanism related to HUA have not been clear. In this paper, UPLC-QE-Orbitrap-MS was used to systematically analyze the components of PS that migrate to the blood; a total of 13 chemical components were identified in the rat serum sample. Notably, the prototype components present in PS, including acteoside, geniposidic acid, and isoacteoside, were detected in rat plasma after intragastric administration of PS extract, and the first two components are also key components for the quality control of medicinal materials. Acteoside and isoacteoside can lower uric acid by inhibiting XOD activity [32,33,34]. Luteolin showed inhibitory activity of XOD; it decreased expression levels of *Urat1* and *Glut9* while elevating organic anion transporter 1 (*Oat1*) and organic anion transporter 3 (*Oat3*) levels, leading to increased uric acid excretion [35,36]. Apigenin ameliorates hyperuricemic nephropathy by inhibiting *Urat1* and *Glut9* and relieving renal fibrosis [35,37]. Apigenin intervention decreased serum UA, XOD, IL-6, IL-1β, IL-18, and TNF-α as well as *Urat1* and *Glut9* levels and increased *Oat1* [38]. Similarly, quercetin is another *Urat1* inhibitor [39]. Geniposidic acid regulates lipid metabolism, inflammation, and oxidative stress and has the potential to improve HUA with abnormal lipid metabolism [40]. Aucubin activates *Pparα*, *Pparγ*, and *Nrf2* to inhibit the production of fat, promote the oxidation of fatty acids, and regulate lipid metabolism [41]. Therefore, PS affects the production of uric acid by inhibiting XOD, and it promotes excretion of uric acid by regulating the uric acid transporter.

In this study, the results demonstrated the favorable therapeutic efficacy of PS in HUA prevention and therapy, manifesting in the amelioration of renal tissue pathology in the presence of HUA. PS lowered the levels of serum uric acid and serum xanthine oxidase (XOD) in model group rats and regulated TNF-α and IL-6 factors. XOD is a key enzyme in the production of uric acid and has a direct impact on the level of uric acid [20]; PS inhibits XOD activity to reduce uric acid and treat gout [34]. Furthermore, high uric acid is often associated with some inflammatory reactions, and lowering the inflammatory response can promote the excretion of uric acid in the kidneys [42,43]. PS has a certain inhibitory effect on the inflammatory response caused by HUA. Studies have shown that high uric acid can increase the expression of inflammatory factors induced by TNF-α in the TNF family and aggravate the inflammatory response [44]. PS effectively reduced TNF-α levels and suppressed the expression of TNF-α-induced inflammatory factors in HUA rats. HUA is closely related to inflammation, lipid metabolism [10], kidney disease, diabetes, etc. [45,46]. As a traditional Chinese medicine, PS enters the kidney channels and has the effects of disinhibiting water, penetrating dampness, and removing phlegm [7]. Reduced uric acid excretion is a key factor in primary hyperuricemia, accounting for about 90% [22]. Uric acid transporters exist widely in renal tubular epithelial cells, and a large number of studies showed that a decrease in renal uric acid excretion was closely related to uric acid transporters [23,47]. PS induced a decrease in the mRNA expression levels of *Urat1* and *Glut9* in the rat kidney model, suggested its potential in promoting uric acid excretion. 

RNA-seq analysis showed that PS is involved in the oxoacid metabolic process, organic acid metabolic process, amino acid transmembrane transporter activity, and lipid metabolic process, suggesting PS’s potential role in lipid metabolic and amino acid metabolism. Earlier metabolomics studies demonstrated that PS treatment might be able to normalize metabolic perturbations caused by HUA. A total of 13 potential biomarkers were identified in HUA rats as being primarily involved in six metabolic pathways, namely the glycerophospholipids metabolism, linoleic acid metabolism, glycosylphosphatidylinositol anchor biosynthesis, alpha-linolenic acid metabolism, arachidonic acid metabolism, and the steroid biosynthesis pathways [13]. Phosphatidylcholine (PC), phosphatidylethanolamine (PE), and LysoPC are mainly involved in glycerophospholipids metabolism, and their levels were significantly increased in HUA rats. PS regulated PC, PE, and LysoPC in relation to HUA [13]. Glycerophospholipids metabolism is a series of catalytic reactions regulating the synthesis and degradation of glycerophosphate, which is closely related to development of metabolic diseases [48,49,50]. Glycerophospholipid metabolism is involved in the process of fat synthesis and degradation [51,52]. 

Combined with RNA-seq results, PS regulates lipid metabolism pathways including the PPAR signaling pathway, fatty acid degradation pathway, retinol metabolism pathway, and fatty acid elongation pathway. The PPAR signaling pathway is directly related to the regulation of lipid metabolism [53], *Pck1, Pparα, Pparγ, Lpl,* and *Plin5* are implicated. *Pck1* is directly involved in gluconeogenesis [54], *Pparα* and *Pparγ* play a key role in adipocyte differentiation and glucose uptake [55,56], and *Lpl* and *Plin5* are involved in the dynamic change of lipid [57,58]. The fatty acid degradation pathway involves the decomposition of fatty acids through β-oxidation, and key genes such as *Cyp4a1*, *Cpt2*, and *Acaa2* are involved. The retinol metabolism pathway involves *Cyp2c23*, *Cyp26b1*, *Cyp4a1* and other enzymes; it is essential for the regulation of lipid homeostasis [59]. Fatty acid elongation is directly involved in modifying the length of fatty acid chains, and *Acot4* and *Acaa2* are involved in this process. It was demonstrated that HUA is a possible risk factor for dysregulation in lipid metabolism [60]. RNA-seq results indicated PS regulates amino acid metabolism pathways, including protein digestion and absorption; glycine, serine, and threonine metabolism; and phenylalanine metabolism pathways. The transporters *Slc7a8*, *Slc6a19*, *Slc16a10*, *Slc38a2*, *Slc15a1*, and *Col1a1* in the protein digestion and absorption pathway affect the absorption of amino acids from dietary proteins and the production of uric acid [61]. The glycine, serine, and threonine metabolism pathway involves the differentially expressed genes *Bhmt, Gatm*, and *Phgdh*; it is essential for the production of nucleotides [62], and the breakdown of nucleotides leads to the formation of uric acid [63]. Our previous studies revealed PS’s capability to alleviate inflammation [13], and the RNA-seq results suggest the mechanisms of PS against HUA are related to the inflammatory mediator regulation of TRP channels and arachidonic acid metabolism pathways. There was no pathway related to adverse drug reactions, such as liver and kidney injury, found in the pathways identified by RNA-seq, and no obvious side effects was observed in the histopathological studies of the current research, indicating that PS has a high safety threshold.

The RT-qPCR results indicated that PS upregulated the mRNA expressions of *Slc7a8*, *Pck1*, *Mgll*, and *Bhmt* and downregulated the mRNA expressions of *Fkbp5*, which are consistent with the results of RNA-seq. *Slc7a8* is associated with hypertension and obesity [25,64,65]. *Pck1* is a gluconeogenic enzyme in the PPAR pathway and holds a well-established role in gluconeogenesis within the liver and kidney, which influences fat production and metabolism and thus is involved in metabolic syndrome [66]. *Pck1* is highly expressed in proximal tubules and is essential for maintaining renal tubule acid–base control, physiology, and lactate and glucose homeostasis. *Bhmt* is cysteine methyltransferase in the glycine, serine, and threonine metabolism pathway, which contributes to the metabolism of amino acids and hepatic gluconeogenesis [27] and affects homocysteine metabolism in the kidney [28]. *Mgll* is an enzyme that converts triglycerides to free fatty acids [26], and it plays an important role in lipid metabolism. *Fkbp5*, a prominent mRNA in the tubulointerstitial compartment [67], has implications for fibrosis [68] and inflammation [29], and PS exerts a downregulating effect on its expression. The results suggest PS’s role in addressing lipid metabolic anomalies triggered by HUA. The PPAR signaling pathway is closely related to lipid metabolism [69], and our study showed that PS regulated *Pparα* and *Pparγ*, *Lpl*, and *Plin5* expression to impact lipid metabolism, further influencing *Urat1* and *Glut9* expression and leading to reduced uric acid reabsorption. Western blotting results further demonstrated the regulation of PS on the expression of URAT1 and PPARα proteins, suggesting that PS regulates lipid metabolism through the PPAR pathway, reducing the levels of uric acid by inhibiting the URAT1. In addition, the single-nucleotide polymorphisms in URAT1 were certified to be associated with the pathogenesis of metabolic syndrome [47]. Clinical studies have found that HUA is associated with hyperlipidemia and is related to lipid metabolism disorders, which can be considered as a risk factor for hyperlipemia and an independent predictive factor [70]. Epidemiological research showed that HUA is significantly correlated with blood lipid abnormalities [71]. The effect of PS on the regulation of uric acid and lipid showed its potential in the treatment of HUA and HUA-induced lipid metabolism abnormalities and metabolic syndrome. PS has a long history of clinical use, and it has few side effects. As a traditional Chinese medicine, PS has multi-component, multi-target, and multi-pathway characteristics. It acts on uric acid transporters to lower uric acid levels, regulates lipid metabolism disorders, and alleviates inflammatory responses. Comprehensive treatment can be carried out from various aspects, demonstrating its potential in the treatment of HUA.

## 4. Materials and Methods

### 4.1. Materials and Reagents

Plant name: Plantaginis semen, the dried, ripe seed of *Plantago asiatica* L. (https://www.worldfloraonline.org/taxon/wfo-0000487709, accessed on 26 July 2024) or *Plantago depressa* Willd. (https://www.worldfloraonline.org/taxon/wfo-0000486802, accessed on 26 July 2024). It is harvested in the summer and autumn when the seeds are ripe. The local name is “Cheqianzi”.

PS was purchased from Beijing Qiancao Traditional Chinese Medicine Slices Co., Ltd. (Beijing, China, NO. 200610005) and identified by Professor Yaojun Yang of Beijing University of Traditional Chinese Medicine as the dried and mature seed of *Plantago asiatica* L. Benzbromarone tablets were produced by Excella GmbH (Nurnberger, Germany). Potassium oxonate was provided by Shanghai Macklin Biochemical Co., Ltd. (Shanghai, China). Acteoside (NO. DSTDL006101), isoacteoside (NO. DST200513-060), geniposidic acid (NO. DST200624-040), luteolin (NO. DST200910-032), quercetin (NO. DSTDH002801), aucubin (NO. DST200706-004), eriodictyol (NO. DSTDS004201), apigenin (NO. DST200809-026), and ursolic acid (NO. DSTDX001901) were all purchased from Chengdu Desite Biotechnology Co., Ltd. (Chengdu, China), with a purity of >98%.

The MS-grade acetonitrile, methanol, and formic acid were obtained from Thermo Fisher Scientific Co., Ltd. (Shanghai, China). Analytical acetonitrile methanol and formic acid were obtained from Tianjin Zhiyuan Chemical Reagent Co., Ltd. (Tianjin, China). Distilled water was purchased from Guangzhou Watsons Food and Beverage Co., Ltd. (Guangzhou, China).

### 4.2. Samples Preparation and Standard Solutions

One kilogram of PS was crushed and passed through a 24-mesh sieve; 65% ethanol of 8 times the amount of medicinal material was added, and it was heated and refluxed 3 times, 2 h each time. The filtrates obtained from three reflux times were mixed. Sample solution: The filtrate was cooled to room temperature and centrifuged at 12,000 rpm for 15 min, yielding the supernatant. Gavage solution: The filtrate was concentrated in a thermostatic water bath at 80 °C to a concentration of 2.0 g/mL of the crude drug and refrigerated for further use.

Standard solution: We took a proper amount of acteoside, isoacteoside, geniposidic acid, luteolin, quercetin, aucubin, eriodictyol, apigenin, and ursolic acid and added methanol to dissolve it, obtaining the single-reference solution. The mixed standard solution was obtained by mixing and diluting each reference solution.

### 4.3. Analysis of PS Components Migrating into the Blood

Twelve healthy Sprague–Dawley rats (male, SPF-grade, 200 ± 20 g, 6 weeks) were purchased from Vital River Laboratory Animal Technology Co., Ltd. (Beijing, China), certificate number: SYXK (jing) 2020-0033. Rats were fed in an animal room with standard laboratory environment (temperature: 20–25 °C, relative humidity: 50–65%, 12 h light and 12 h dark daily to simulate the diurnal cycle). The rats were fed adaptively for 3 days and then randomly divided into 2 groups (n = 6), including the control group (group A) and the PS-administration group (group B). In group B, the gavage solution was given intragastrically for three consecutive days according to weight (30 g/kg), and the rats were fasted for 12 h before the last administration. Group A was given the same dose of distilled water. In group B, the plasma samples were collected from the fundus vein of rats at 15 min, 30 min, 1 h, 1.5 h, 2 h, 4 h, and 6 h after administration, respectively. The obtained plasma samples were centrifuged at 3500 rpm and 4 °C for 10 min, and the supernatant was stored at –80 °C for later use. 

Then, 50 μL of plasma was collected from each group at different blood collection points and combined with 3 times the amount of acetonitrile, vortexed for 5 min, and centrifuged at 12,000 rpm for 15 min at 4 °C. The supernatants were taken and dried with nitrogen, and the residue was added to 400 μL 50% methanol, vortexed for 5 min, dissolved, and centrifuged at 12,000 rpm for 15 min, after which the supernatants were collected for subsequent analysis. 

### 4.4. Qualitative Analysis by UPLC-QE-Orbitrap-MS

Qualitative analysis was conducted using an ACQUITY UPLC BEH C_18_ Column (2.1 mm × 100 mm, 1.7 μm, Waters Corp., MA, USA) with a temperature of 30 °C by UPLC (Thermo Fisher Ultimate 3000, MA, USA) coupled with a QE-Orbitrap mass spectrometer (Thermo Fisher Ultimate, MA, USA). The mobile phase was composed of 0.1% (volume fraction) formic acid aqueous solution (A)-methanol (B) with a gradient elution procedure as follows: 0~1 min, 2% B; 1~4 min, 2%~30% B; 4~12 min, 30% B; 12~13 min, 30~40% B; 13~15 min, 40% B; 15~17 min, 40~60% B; 17~23 min, 60~66% B; 23~24 min, 66% B; 24~30 min, 66~98% B; 30~31 min, 98% B; 31~32 min, 98~2% B; and 32~33 min, 2% B. The flow rate was set at 0.25 mL/min, the sample chamber temperature was maintained at 4 °C, and the injection volume was 2 μL.

In mass spectrometry, the electric spray ionization source (ESI) was employed in both negative and positive modes, and the MS conditions used for analysis were as follows: the scanning mode was full scan/data-dependent secondary scan (dd-MS^2^) and covered the mass-to-charge (*m*/*z*) range from 100 to 1200 with a full scan at a primary MS resolution of 70,000 and dd-MS^2^ resolution of 17,500; the capillary temperature was 350 °C, the spray voltage of positive-ion mode was 3.5 kV, the spray voltage of negative-ion mode was 2.5 kV, the sheath gas flow rate was 35 arb (arbitrary units), and the aux gas flow rate was 15 arb; MS^2^ adopted three kinds of collision energy: 30 V, 50 V, and 70 V. 

### 4.5. HUA Rat Model Establishment and Drugs Administration 

Male Sprague–Dawley rats (220 ± 20 g, SPF, 6–7 weeks, certificate number: SCKY (jing) 2021-0011) were purchased from Beijing Vitong Lihua Experimental Animal Technology Co., Ltd. (Beijing, China). The rats were maintained in a room with a controlled temperature (20–25 °C). All experimental animals were kept for a 3-day acclimation period before the experiments. All experimental procedures and protocols were reviewed and approved by the Animal Care and Use Committee of the Beijing University of Chinese Medicine (Approval number: BUCM-2023051103-2225). All experiments were performed in accordance with the approved Regulations on the Management of Experimental Animals and the Detailed Rules on the Management of Chinese Medical Experimental Animals. 

The HUA rat model was established by intragastric administration of potassium oxonate for 28 days [72,73]. The success of the model was judged by serum uric acid, serum xanthine oxidase levels, and the pathological changes in the kidney of rats [74,75,76]. Thirty-two rats were randomly divided into four groups (n = 8): the control group (control), model group (model), benzbromarone group (BBR), and Plantaginis semen group (PS). At 1 h before administration, rats were given potassium oxonate by intragastric administration at a dose of 1.5 g/kg. Administration and modeling induction were carried out simultaneously for 28 days. The control group was given the corresponding volume of distilled water by intragastric administration. The PS group was given PS at a dosage of 3.75 g/kg by intragastric administration. The BBR group was given benzbromarone (10 mg/kg). The dosage of PS is detailed in the previous study and was explored in the early stage. As can be seen in the reference, the dosages of 0.9375, 1.875 and 3.75 g/kg were compared, and the group with a dosage of 3.75 g/kg had the best effect on lowering uric acid [13]. The animals fasted overnight on the last day of the experimental model, and then anesthesia was induced and samples collected. After 28 days, the blood and kidneys (one kidney was fixed with 4% paraformaldehyde fixation and the other was stored at −80 °C) were collected. The blood samples were centrifuged at 3500 rpm for 15 min, and the supernatants were collected and refrigerated at −80 °C. 

### 4.6. Biochemical Index Detection 

An enzyme-linked immunosorbent assay was used to detect UA, XOD, TNF-α, and IL-6 in serum, and the experiments were conducted in strict accordance with the instructions by microplate reader (BioTek Epoch, Santa Clara, CA, USA). ELISA kits were purchased from Jiangsu Kete Biotechnology Co., Ltd. (Xuzhou, Jiangsu, China)

### 4.7. Hematoxylin–Eosin (H&E) Staining

Paraffin-embedded kidney slices (4 µm) were placed in a 60 °C incubator and baked. Then, the slides were immersed for dewaxing (xylene I 20 min → xylene II 20 min → Anhydrous ethanol Ⅰ 10 min → anhydrous ethanol II 10 min → 95% alcohol 5 min → 90% alcohol 5 min → 80% alcohol 5 min → 75% alcohol 5 min → distilled water). Then, the slices were dyed in hematoxylin for 5 min, washed with tap water, put into 1% hydrochloric acid alcohol solution for a few seconds, rinsed with tap water, put into 0.6% ammonia water to blue, and rinsed with running water again. Then, the slices were kept in an eosin dyeing solution and stained for 2 min. After dehydration and xylene removal twice, the tissue sections were sealed with neutral gum and manually scanned with a light microscope (Nikon Corporation, Tokyo, Japan).

### 4.8. RNA Sequencing

Renal tissue samples were used to extract total RNA, which was then analyzed for concentration and purity using Nanodrop 2000 (Thermo Fisher Scientific Co., Ltd.). Magnetic beads and Oligo (dT) were used to enrich mRNA. By adding the fragmentation buffer, mRNA was randomly fragmented, and small fragments of about 300 bp were enriched by magnetic bead. Under the action of reverse transcriptase, six-base random hexamers were added to invert the mRNA template to synthesize a strand cDNA, and a stable double-strand structure was then formed by double-strand synthesis. The SuperScript double-stranded cDNA synthesis kit (Invitrogen, Waltham, CA, USA) and random hexamer primers (Illumina, San Diego, CA, USA) were used. In accordance with Illumina’s library construction protocol, the synthesized cDNA was subjected to end-repair, phosphorylation, and “A” base addition and sequenced using the Illumina Novaseq 6000 platform. 

To identify DEGs between two samples, the transcripts per million reads method was used to calculate the expression levels of each transcript. Gene abundance was quantified using RSEM [77], and DESeq2 [78] was used to determine DGEs based on |log2 (Fold Change)| ≧ 1 and FDR ≤ 0.05 (DESeq2) or FDR ≤ 0.001 (DEGseq). Then, functional enrichment analyses including GO and KEGG were performed by Goatools and KOBAS [77], respectively.

### 4.9. Real-Time Quantitative PCR 

RT-qPCR was performed to detect the transcription levels of gene mRNAs in renal tissue samples. Total RNA was isolated from kidney tissues using FastPure^®^ Cell/Tissue Total RNA Isolation Kit (RC101, Vazyme, Nanjing, China), and RNA concentration and purity were measured by spectrophotometry. RNA was reverse-transcribed using the ABScript III RT Master Mix (RK20429, ABclonal, Wuhan, China). RT-qPCR was performed by Bio-Rad Real-time PCR (CFX96 Touch, Bio-Rad, Hercules, CA, USA) and 2X Universal SYBR Green Fast qPCR Mix (RK21203, ABclonal, Wuhan, China). Relative gene expression was calculated using the 2^−∆∆Ct^ method with *Gapdh* as an internal control. The primer sequences reported previously [79,80,81,82,83,84,85,86,87,88,89,90,91] were used in the current study (Table 3).

### 4.10. Western Blotting

Rat kidney tissues were lysed with RIPA buffer containing phosphatase and protease inhibitors, and total protein was quantified using a BCA kit (A045-4-2, Nanjing, China). After denaturation at 100 °C for 5 min, the protein was diluted with 3 times the loading buffer for SDS-PAGE electrophoresis and then transferred to PVDF membranes and blocked with TBST containing 5% milk powder for 1 h with gentle shaking. The PVDF membrane was incubated at 4 °C overnight with primary antibodies as follows: URAT1 (61 kD, Cat# YN3803, 1:1000, Immunoway), PPARα (52 kD, Cat# YT3835, 1:1000, Immunoway, Plano, TX, USA), and GAPDH (38 kD, Cat# YM3029, 1:1000, Immunoway, TX, USA). Then, it was incubated with secondary goat anti-rabbit IgG (H+L) [86 RS0002, 1:5000, Immunoway] or goat anti-mouse IgG (H+L) [86 RS0001, 1:5000, Immunoway] at room temperature. The PVDF membrane blot was developed using the ChemiDoc imaging system (ChemiDoc MP, Bio-rad, Hercules, CA, USA) and analyzed using ImageJ software (version 1.54j).

### 4.11. Statistical Analysis

The in vitro data are presented as mean ± standard deviation (SD). Significance was analyzed using GraphPad Prism 6.0 (GraphPad Software Inc., Boston, MA, USA) through one-way ANOVA. * *p* < 0.05, ** *p* < 0.01, and *** *p* < 0.001 are considered statistically significant.

## 5. Conclusions

In this study, UPLC-QE-Orbitrap-MS and multi-omics techniques were employed to identify the bioactive components of PS in vitro and in vivo, and the serum UA, XOD, and cytokine levels confirmed the efficacy of PS in prevention of HUA. The efficacy of PS for HUA mainly involves the oxoacid metabolic process, organic acid metabolic process, amino acid transmembrane transporter activity, and lipid metabolic process. PS is involved in regulating lipid metabolism, amino acid metabolism, and inflammatory response. PS upregulated mRNA levels of *Slc7a8*, *Pck1*, *Mgll*, and *Bhmt* while downregulating the mRNA expression of *Fkbp5*. These observations suggest that PS activates the PPAR signaling pathway and regulated uric acid transporters. These findings pave solid foundations for illustrating the anti-HUA mechanisms of PS and clarifying potential active ingredients, opening the path for further investigation of PS for HUA.

## Figures and Tables

**Table 1:** Constituents analysis in Plantaginis semen using UPLC-QE-Orbitrap-MS.

NO.	t_R_/min	Formula	Adducts	Theoretical Value	Measured Value	Mass Error (ppm)	MS/MS	Identification	Label
1	1.02	C_6_H_14_O_6_	+H	183.0863	183.0864	0.55	147.1855, 98.9845	Sorbitol	h
2	2.81	C_6_H_8_O_7_	-H	191.0186	191.0201	7.85	191.0199, 173.0089, 111.0089, 87.0088	Citric acid	f
3 *	4.74	C_15_H_22_O_9_	-H	345.1180	345.1195	4.35	299.8194, 139.0407, 89.3671	Aucubin	c
4	5.60	C_16_H_22_O_10_	+H	375.1286	375.1279	−1.87	287.0074, 195.0659, 177.0552, 167.0707, 149.0602, 121.0652	Gardoside	c
5	5.60	C_16_H_20_O_9_	+H	357.1180	357.1175	−1.40	195.0659, 177.0553, 149.0603, 121.0652	Feruloylsucrose	i
6 *	5.61	C_16_H_22_O_10_	-H	373.1129	373.1135	1.61	300.3118, 211.0611, 193.0512, 167.0711, 149.0609, 123.0453, 121.0660	Geniposidic acid	c
7	5.81	C_20_H_30_O_12_	-H	461.1654	461.1672	3.90	461.1658, 315.1101, 161.0455, 135.0452	Verbasoside	b
8	6.30	C_9_H_12_O_4_	+H	185.0808	185.0809	0.54	185.1539, 139.0766, 129.9802, 111.0751	Pedicularis-lactone	c
9	7.46	C_10_H_10_O_4_	-H	193.0495	193.0500	2.59	193.0509, 116.9284, 96.9601	Ferulic acid	f
10	9.50	C_29_H_36_O_16_	-H	639.1920	639.1956	5.63	141.5413	Plantagoamidinic acid	e
11	11.15	C_11_H_19_N_3_O_2_	-H	224.1394	224.1405	4.91	224.1392, 141.0924	Plantagoamidinic acid A	e
12	11.72	C_21_H_22_O_12_	-H	465.1028	465.1042	3.01	465.1032, 303.0506, 151.0393	Plantagoside	a
13	14.23	C_29_H_36_O_16_	-H	639.1920	639.1954	5.32	477.1617, 179.0362, 161.0246, 135.0457	Plantamajoside	b
14	15.12	C_15_H_12_O_7_	-H	303.0499	303.0514	4.95	303.0510, 151.0402, 107.0139	Taxifolin	a
15*	15.41	C_29_H_36_O_15_	-H	623.1970	623.2000	4.81	623.1987, 461.1670, 315.1082, 179.0352, 161.0246, 153.0559, 135.0453	Acteoside	b
16	15.41	C_29_H_34_O_15_	-H	621.1814	621.1828	2.25	179.0350, 161.0246, 135.0453	Orobanchoside	b
17	15.94	C_23_H_26_O_11_	-H	477.1391	477.1410	3.98	477.1409, 315.1057, 161.0234, 135.0448	Calceolarioside B	b
18	16.34	C_21_H_20_O_11_	-H	447.0922	447.0941	4.25	447.0935, 285.0405, 284.0324, 256.0373, 199.0396, 151.0041	Cynaroside	a
19	16.45	C_29_H_36_O_16_	-H	639.1920	639.1954	5.32	639.1927, 477.1605, 315.1107, 179.0350, 161.0246, 153.0549, 135.0454, 84.7942	Plantainoside D	b
20	17.10	C_27_H_30_O_15_	-H	593.1501	593.1522	3.54	285.0384, 284.0322	Kaempferol-3-rutinoside	a
21 *	17.15	C_29_H_36_O_15_	-H	623.1970	623.2000	4.81	623.1990, 461.1681, 179.0350, 161.0245, 135.0453	Isoacteoside	b
22	17.58	C_27_H_32_O_14_	-H	579.1708	579.1725	2.94	271.0618, 151.0029	Naringin	a
23 *	17.62	C_15_H_12_O_6_	+H	289.0707	289.0704	−1.04	271.0609, 179.0344, 163.0395, 153.0188	Eriodictyol	a
24	17.87	C_21_H_20_O_12_	+H	465.1028	465.1031	0.65	302.0745, 300.3060	Hyperoside	a
25	17.96	C_21_H_20_O_10_	-H	431.0973	431.0994	4.87	431.0989, 269.0454, 268.0375, 151.0038	Apigenin 7-glucoside	a
26	18.11	C_27_H_30_O_14_	-H	577.1552	577.1575	3.99	413.0989, 269.0454, 268.0375, 183.0457	Rhoifolin	a
27	18.26	C_31_H_38_O_16_	-H	665.2076	665.2112	5.41	503.1779, 181.5070, 161.0246	2-Acetylacteoside	b
28	18.80	C_21_H_20_O_11_	+H	449.1078	449.1080	0.45	449.0978, 287.0555	Plantaginin	b
29 *	18.91	C_15_H_10_O_7_	-H	301.0343	301.0358	4.98	301.0358, 254.8575, 121.6543	Quercetin	a
30	19.27	C_22_H_22_O_11_	-H	461.1078	461.1094	3.47	461.1952, 181.7875	Homoplantaginin	a
31 *	19.90	C_15_H_10_O_6_	-H	285.0394	285.0408	4.91	285.0408, 199.0405, 175.0404, 151.0036, 133.0297, 107.0138, 83.0142	Luteolin	a
32 *	21.05	C_15_H_10_O_5_	-H	269.0444	269.0456	4.46	269.0456, 200.9979, 117.0348, 65.0031	Apigenin	a
33	24.74	C_15_H_10_O_5_	-H	269.0444	269.0456	4.46	269.0456, 241.0506, 225.0557, 197.0611	Baicalein	a
34	29.15	C_9_H_8_O_4_	+H	181.0495	181.0493	−1.10	181.0718, 163.0395, 135.0446, 116.9860, 79.0545, 65.0390	Caffeic acid	f
35	29.77	C_30_H_48_O_4_	-H	471.3469	471.3484	3.18	471.3484, 453.3378, 427.3596, 407.3329	Maslinic acid	d
36	31.03	C_18_H_32_O_2_	+H	181.0495	181.0493	−1.10	181.0718, 163.0395, 135.0446, 116.9860, 79.0545, 65.0390	Caffeic acid	f
37	31.39	C_30_H_48_O_3_	-H	455.3520	455.3535	3.29	456.3588, 455.3534, 408.2341	Oleanic acid	d
38	31.58	C_18_H_30_O_2_	-H	277.2162	277.2173	3.97	277.2173, 259.2071, 233.1541, 121.0296, 81.9533, 59.0138	α-Linolenic acid	g
39 *	31.95	C_30_H_48_O_3_	-H	455.3520	455.3533	2.85	455.3536, 300.0453, 181.7717, 147.1723, 115.9207, 99.9257	Ursolic acid	d

Note: The * in the NO. column indicates compounds that have been compared with standards; a denotes flavonoids, b denotes phenyl ethanol glycosides, c denotes iridoids, d denotes triterpenoids, e denotes alkaloids, f denotes organic acids, g denotes fatty acids, h denotes glycitols, and i denotes phenylpropanoid.

**Table 2:** Analysis of blood-migrating components of Plantaginis semen using UPLC-QE-Orbitrap-MS.

NO.	t_R_/min	Formula	Adducts	Theoretical Value	Measured Value	Mass Error (ppm)	MS/MS	Identification
1	2.53	C_6_H_8_O_7_	-H	191.0186	191.0199	6.81	191.0200, 173.0090, 111.0088, 87.0088	Citric acid ^a^
2	3.51	C_9_H_13_NO_9_S	-H	310.0227	310.0247	6.45	310.0974, 230.0188, 87.1158	Citric acid ^j^
3	4.72	C_15_H_22_O_9_	-H	345.1180	345.1193	3.77	299.9364, 139.0401, 89.3645	Aucubin ^a^
4	4.79	C_22_H_30_O_16_	-H	549.1450	549.1460	1.82	211.0613, 167.0714, 149.0607, 123.0452, 121.0660	Geniposidic acid ^i^
5	4.96	C_16_H_22_O_13_S	-H	453.0697	453.0713	3.53	453.0710, 300.4093, 193.0514, 149.0610, 123.0451, 121.4576	Geniposidic acid ^h^
6	5.60	C_16_H_22_O_10_	-H	373.1129	373.1135	1.61	211.0614, 193.0512, 167.0714, 149.0609, 123.0452, 121.0660	Geniposidic acid ^a^
7	6.09	C_15_H_20_O_9_	-H	343.1024	343.1037	3.79	343.1034, 181.6917, 162.8395, 160.8422, 159.8602, 139.0766, 101.0247, 99.0089, 89.0247	Aucubin ^d^
8	6.42	C_15_H_20_O_8_	-H	327.1074	327.1089	4.59	165.0558, 119.1114, 99.0086, 89.0245	Aucubin ^b^
9	6.92	C_16_H_20_O_9_	-H	355.1024	355.1038	3.94	300.2832, 121.0297	Geniposidic acid ^b^
10	7.11	C_10_H_10_O_4_	-H	193.0495	193.0507	6.22	193.8154, 149.0606, 133.0296	Ferulic acid ^a^
11	11.59	C_11_H_19_N_3_O_2_	-H	224.1394	224.1406	5.35	224.1406, 141.0923	Plantagoamidinic acid A ^a^
12	12.33	C_21_H_20_O_12_	-H	463.0871	463.0890	4.10	151.0039, 107.0139	Plantagoside ^d^
13	15.33	C_29_H_36_O_15_	-H	623.1970	623.2006	5.78	623.2032, 461.1672, 179.0350, 161.0245, 135.0452	Acteoside ^a^
14	15.77	C_21_H_20_O_11_	-H	447.0922	447.0939	3.80	447.1942, 151.0037, 107.0140	Plantagoside ^b^
15	15.77	C_21_H_20_O_11_	-H	447.0922	447.0939	3.80	447.1942, 151.0037	Cynaroside ^a^
16	17.04	C_29_H_36_O_15_	-H	623.1970	623.2005	5.62	623.2021, 461.1671, 161.0244, 135.0457	Isoacteoside ^a^
17	17.90	C_21_H_18_O_11_	-H	445.0765	445.0783	4.04	445.1906, 269.0458	Cynaroside ^d^
18	17.90	C_21_H_18_O_11_	-H	445.0765	445.0783	4.04	445.1906, 269.0458, 117.0347, 65.3859	Apigenin ^i^
19	17.94	C_21_H_22_O_11_	-H	449.1078	449.1094	3.56	258.0529, 273.0766, 151.0039	Cynaroside ^e^
20	18.76	C_24_H_40_O_8_	+H	457.2796	457.2792	−0.87	457.2742, 281.0787, 133.0863	Linoleic acid ^i^
21	18.83	C_21_H_18_O_12_	-H	461.0715	461.0732	3.69	461.1859, 285.0403, 199.0399, 175.0396, 151.0038, 133.0296, 107.0138	Luteolin ^i^
22	19.13	C_15_H_10_O_9_S	-H	364.9962	364.9978	4.38	365.1043, 213.0549, 175.0405, 151.0033	Luteolin ^h^
23	19.27	C_22_H_22_O_11_	-H	461.1078	461.1094	3.47	461.1850, 285.0768, 165.1093, 151.0033	Cynaroside ^g^
24	19.27	C_22_H_22_O_11_	-H	461.1078	461.1094	3.47	461.1850, 181.7875	Homoplantaginin ^a^
25	19.31	C_15_H_12_O_5_	-H	271.0601	271.0613	4.43	271.1891, 227.1645, 151.0042, 119.0504, 116.9956, 65.6944	Apigenin ^e^
26	19.77	C_15_H_10_O_6_	-H	285.0394	285.0406	4.21	285.0402, 194.9880, 166.9930, 133.0293, 116.9960	Apigenin ^f^
27	20.89	C_15_H_10_O_5_	-H	269.0444	269.0452	2.97	269.1378, 225.1501, 200.9967, 182.9875, 116.9956	Apigenin ^a^
28	23.19	C_20_H_34_O_3_	+H	323.2581	323.2575	−1.86	323.2375, 149.1331, 133.1017, 107.0858, 97.0651, 69.0703, 55.0547	Linoleic acid ^k^
29	29.09	C_9_H_8_O_4_	+H	181.0495	181.0494	−0.55	181.1596, 163.0395, 135.0445, 116.9861, 79.0545, 65.0390	Caffeic acid ^a^
30	29.91	C_18_H_30_O_3_	-H	293.2111	293.2122	3.75	293.2117, 275.2018, 249.2219, 121.1017, 59.0137	α-Linolenic acid ^f^
31	29.95	C_18_H_32_O_3_	-H	295.2268	295.2278	3.39	295.2278, 277.2172, 121.5115, 59.0137	α-Linolenic acid ^c^
32	29.96	C_18_H_30_O_2_	+H	279.2319	279.2313	−2.15	279.2327, 261.2224, 173.1333, 165.1279, 163.1124, 133.1024, 131.0859, 107.0857, 105.0704, 67.0546, 65.0388, 55.0548, 53.0390	Linoleic acid ^d^
33	30.27	C_18_H_32_O_3_	+H	297.2424	297.2420	−1.35	279.2326, 191.1441, 181.1586, 165.1280, 149.1332, 133.1015, 123.1173, 107.0859, 69.0702, 55.0548	Linoleic acid ^f^
34	30.42	C_18_H_30_O	+H	263.2369	263.2365	−1.52	263.2372, 245.2271, 175.1492, 165.1640, 133.1016, 107.0859, 69.0702, 55.0548	Linoleic acid ^b^
35	30.45	C_36_H_56_O_9_	-H	631.3841	631.3865	3.80	456.3566, 455.3532, 408.2441	Oleanolic acid ^i^
36	31.07	C_18_H_28_O_2_	-H	275.2006	275.2015	3.27	275.2012, 231.2118, 121.7366, 79.9572, 59.0139	α-Linolenic acid ^d^
37	31.52	C_18_H_30_O_2_	-H	277.2162	277.2171	3.25	277.2171, 259.2079, 233.0141, 59.0137	α-Linolenic acid ^a^
38	31.95	C_30_H_48_O_3_	-H	455.3520	455.3537	3.73	455.3168, 300.1568, 181.5451, 115.9202, 99.9258	Ursolic acid ^a^
39	32.01	C_18_H_32_O_2_	-H	279.2319	279.2328	3.22	279.2328, 261.2221, 121.6766, 59.0138	α-Linolenic acid ^e^
40	32.50	C_18_H_34_O_2_	+H	283.2632	283.2625	−2.47	177.1648, 135.1137, 109.1016, 107.0860, 71.0859, 69.0702, 57.0704, 55.0548	Linoleic acid ^e^

^a^: prototype; ^b^: dehydration; ^c^: hydrolysis; ^d^: dehydrogenation; ^e^: hydroreduction; ^f^: hydroxylation; ^g^: methylation; ^h^: sulfation; ^i^: conjugated glucuronic acid; ^j^: conjugated cysteine; ^k^: acetylation.

**Table 3:** Nucleotide sequences of RT-qPCR primers.

Genes	Forward Primer (5′ to 3′)	Reverse Primer (5′ to 3′)
*Urat1*	CTCTGCCTTTCTCCTGTTGA	CCCCTTGATGATGACCTTG
*Glut9*	TTCGGGTCCTCCTTCCTCTA	GGACACAGTCACAGACCAGA
*Slc7a8*	TCCACATTTGGTGGAGTCAA	TGGATCATGGCTAACACGCT
*Bhmt*	CGGGCAGACCGTACAATCC	CTTTCGTCACTCCCCAAGCA
*Fkbp5*	GAACCCAATGCTGAGCTTATG	ATGTACTTGCCTCCCTTGAAG
*Mgll*	GCGGTGCCATCTCCATCC	GCTCCTGCCACTGCTATCC
*Pparα*	TCACACAATGCAATCCGTTT	GGCCTTGACCTTGTTCATGT
*Pparγ*	CATGCTTGTGAAGGATGCAAG	TTCTGAAACCGACAGTACTGACAT
*Lpl*	AGACTCGCTCTCAGATGCCCTAC	TCACTTTCAGCCACTGTGCCATAC
*Plin5*	AAATCAGAGGAGCTGGTGGA	ACGCACAAAGTAGCCCTGTT
*Atgl*	CCCTGACTCGAGTTTCGGAT	CACATAGCGCACCCCTTGAA
*Hsl*	GCCAGCCACAACCTAGCAGAAC	CATCGCCCTCAAAGAAGAGCACTC
*Gapdh*	TCTCTGCTCCTCCCTGTTC	ACACCGACCTTCACCATCT

## Data Availability

The raw data supporting the conclusions of this article will be made available by the authors on request.

## Abbreviations

*Acaa2*	Acetyl-CoA acyltransferase 2
*Acot4*	Acyl-CoA thioesterase 4
*Atgl*	Adipose triglyceride lipase
*Bhmt*	Betaine-homocysteine S-Methyltransferase
*Col1a1*	Collagen Type I Alpha 1 Chain
*Cpt2*	Carnitine palmitoyltransferase 2
DEGs	Differential expression genes
DEMs	Differentially expressed metabolites
ELISA	Enzyme-linked immunosorbent assay
*Fkbp5*	FKBP prolyl isomerase 5
H&E	Hematoxylin–eosin
*Hsl*	Hormone-sensitive lipase
HUA	Hyperuricemia
*Gatm*	Glycine amidinotransferase
*Glut9*	Glucose transporter 9
IL-1β	Interleukin-1β
IL-6	Interleukin-6
*Lpl*	Lipoprotein lipase
*Mgll*	Monoacylglycerol lipase
*Pck1*	Phosphoenolpyruvate carboxykinase 1
*Phgdh*	3-Phosphoglycerate dehydrogenase
PI3K	Phosphatidylinositol 3-kinase
*Plin5*	Perilipin-5
PS	Plantaginis semen
PPAR	Peroxisome proliferators-activated receptors
RNA-seq	RNA sequencing
RT-qPCR	Real-time quantitative PCR
*Slc7a8*	Solute carrier family 7 member 8
TGF-β1	Transforming growth factor beta 1
TNF	Tumor necrosis factor
UA	Uric acid
UPLC-QE-Orbitrap-MS	Ultra-high-performance liquid chromatography–tandem quadrupole electrostatic field–orbital trap–mass spectrometry
Urat1	Urate anion transporter 1
XOD	Xanthine oxidase
